# *KRAS*, *NRAS* and *BRAF* mutations detected by next generation sequencing, and differential clinical outcome in metastatic colorectal cancer (MCRC) patients treated with first line FIr-B/FOx adding bevacizumab (BEV) to triplet chemotherapy

**DOI:** 10.18632/oncotarget.25180

**Published:** 2018-05-29

**Authors:** Gemma Bruera, Francesco Pepe, Umberto Malapelle, Pasquale Pisapia, Antonella Dal Mas, Daniela Di Giacomo, Giuseppe Calvisi, Giancarlo Troncone, Enrico Ricevuto

**Affiliations:** ^1^ Oncology Territorial Care, S. Salvatore Hospital, Oncology Network ASL1 Abruzzo, University of L'Aquila, L'Aquila, Italy; ^2^ Department of Biotechnological and Applied Clinical Sciences, University of L’Aquila, L’Aquila, Italy; ^3^ Department of Public Health, University Federico II, Napoli, Italy; ^4^ Pathology, S. Salvatore Hospital, Oncology Network ASL1 Abruzzo, L'Aquila, Italy

**Keywords:** FIr-B/FOx intensive first line triplet chemotherapy plus bevacizumab, metastatic colorectal cancer, next generation sequencing, RAS/BRAF mutations, 50 genes panel

## Abstract

**Background:**

First line triplet chemotherapy/BEV significantly improved clinical outcome of MCRC. *KRAS/NRAS/BRAF* mutations were evaluated by next generation sequencing (NGS) in MCRC patients treated with first line FIr-B/FOx.

**Methods:**

*KRAS* exons 2-4 (*KRAS*_*2-4*_), *NRAS*_*2-4*_, *BRAF*_*15*_ were evaluated in 67 tumours by ION Torrent platform. Mutation detection criteria: >500×sequence coverage (cov); >1% mutant allelic fraction (AF). Clinical outcomes were compared by log-rank.

**Results:**

In 63 samples, *KRAS*_*2-4*_/*NRAS*_*2-4*_/*BRAF*_*15*_ wild-type (wt) were 14 (22.2%), mutant (mut) 49 (77.8%): *KRAS*_*2-4*_ 42 (66.7%); *NRAS*_*2-4*_ 11 (16.4%); *BRAF*_*15*_ 5 (7.5%). Sixty mutations were detected, range 1-3 mut: 43 (71.7%) >1000×cov/>5% AF; 9 (15%) >500×cov/>5% AF; 8 (13.3%) >1000×cov/<5% AF. Mut distribution in *KRAS*_*2-4*_/*NRAS*_*2-4*_/*BRAF*_*15*_: 40 (63.5%) >1000×cov/>5% AF, 8 (12.7%) >500×cov/>5% AF, 1 (1.6%) >1000×cov/<5% AF; *BRAF*_*15*_ 1 (1.5%) >500×cov/>5% AF, 4 (6%) >1000×cov/<5% AF. Prevalence of ≥2 mut samples: *KRAS*_*2-4*_/*NRAS*_*2-4*_/*BRAF*_*15*_ 8 (12.7%); *KRAS*_*2-4*_ 7 (11.1%); *NRAS*_*2-4*_ 5 (7.5%). *BRAF*_*15*_ mutant were all ≥2 mut (7.5%), atypical and associated to *KRAS* and/or *NRAS* mut: c.1405 G>A; c.1406 G>C; c.1756 G>A, 2 samples; c.1796 C>T. At 21 months (m) follow-up, clinical outcome wt compared to mut was not significantly different: in *KRAS*_*2-4*_/*NRAS*_*2-4*_/*BRAF*_*15*_, progression-free survival (PFS) 18/12 m, overall survival (OS) 28/22 m; 1/≥2 mutations, PFS 14/11, OS 37/22. PFS was trendy worse in *RAS*/*BRAF* wt vs ≥2 mut genes (*P* 0.059).

**Conclusions:**

Most MCRC harboured *KRAS*_*2-4*_/*NRAS*_*2-4*_/*BRAF*_*15*_ mutations by NGS, often multiple and affecting few tumoral clones; 22% were triple wt. Clinical outcome is not significantly affected by *KRAS*_*2-4*_/*NRAS*_*2-4*_/*BRAF*_*15*_ genotype, trendy different in triple wt, compared with *KRAS*_*2-4*_/*NRAS*_*2-4*_/*BRAF*_*15*_ ≥2 mut.

## INTRODUCTION

Gain-of-function mutations of *KRAS, NRAS*, *BRAF*, *PIK3CA* genes, or loss of tumor suppressor function of PTEN, resulting in continuous activation of RAS-mitogen-activated protein kinase (MAPK) or phosphoinositide 3-kinase (PI3K) pathways, characterize most colorectal cancers (CRC) [1]. More, 12–15% CRC show high mutational load and microsatellite instability [2]. Specific mutations of the different genes involved in MAPK/PI3K pathway may confer different biological aggressiveness and effectiveness of treatment strategies.

Overall, *KRAS* exons 2–4 (*KRAS*_*2-4*_), *NRAS* exons 2–4 (*NRAS*_2–4_), *BRAF* exon 15 (*BRAF*_15_) mutant (mut) MCRC patients are prevalent [3–6]. *KRAS*_*2*_ mut characterize 45–55% MCRC, mostly consisting of codon 12 (80%) c.35 G>A (G12D) and c.35 G>T (G12V) transversions [7, 8], and codon 13, prevalently c.38 G>A (G13D) mut, and impair the intrinsic GTPase activity of RAS, leading to constitutive, growth-factor-receptor independent activation of downstream signaling [9–11]. In MCRC patients, the reported prevalence of *KRAS*_*2–4*_ mut is 52.8%*, NRAS*_*2–4*_ mut 5.3%, *BRAF*_15_ mut 4.7–8.7%, prevalently c.1799 T>A (V600E) [3, 12]. Massive parallel sequencing of multiple genes by different NGS platforms enables mutation detection highly accurate and it is able to detect mutations at 5% allelic fraction (AF) [13, 14]. PGM/Colon Lung Cancer Panel identified all point mutations, and failed in 4.4% CRC mutant samples. In early CRC, *KRAS*_*2–4*_ 37.8%, and *NRAS*_*2–4*_ 4.6% mut were reported [13, 14]. *KRAS*, *NRAS* mut were mutually exclusive, *BRAF* mut (mostly not V600E) occasionally, *PIK3CA* mut frequently coexisted with *RAS* mut. Detection of multiple gene mutations and dynamic molecular characterization in the individual patient, could help monitoring biological evolution of metastatic disease, with prognostic and predictive clinical implications.

Treatment strategy of MCRC patients differs according to patient’s fitness (age, comorbidity), metastatic extension (liver-limited (L-L) or other/multiple metastatic sites (O/MM)), and *KRAS*_*2–4*_/*NRAS*_*2–4*_/*BRAF*_*15*_ genotype [9, 6, 15]. *KRAS*_*2–4*_/*NRAS*_*2–4*_ wt or mut genotype addresses the addiction of anti-Vascular Endothelial Growth Factor (VEGF) or anti-Epidermal Growth Factor Receptor (EGFR) to first line triplet or doublet chemotherapy of MCRC [3, 16–18]: anti-EGFR drugs in *RAS* wt [16–18]; anti-VEGF drugs in *RAS* wt and mut patients [3]. *KRAS* wt genotype predicted favourable clinical outcomes of anti-EGFR or anti-VEGF molecules added to doublet chemotherapy [19].

Clinical outcome of MCRC patients treated with BEV-containing chemotherapy is not significantly affected by *KRAS*_*2*_ status, wt or mut; median OS ranges between 29.9–38 months, and 19.9–21 months, respectively [19, 20, 9]. In *KRAS*_*2*_ mut patients, retrospective analysis showed that BEV addition to irinotecan, 5-fluorouracil, leucovorin (IFL) was predictive of significantly prolonged PFS, compared to IFL [19, 20]; in *KRAS*_*2*_ wt and mut MCRC patients treated with BEV added to IFL, median OS was 27.7 and 19.9 months, respectively, not significantly different [19, 20]. The prognostic relevance of *KRAS*_*2*_ and *BRAF*_*15*_ genotype was not significantly different, even though the hazard ratio (HR) was 0.64 and 0.38, respectively. A significantly better prognosis was reported only when *KRAS*_*2*_/*BRAF*_*15*_ wt patients were compared with patients harboring mutations in *KRAS*_*2*_ or *BRAF*_*15*_ genes (HR 0.51) [19]. Intensive first line treatment adding BEV to triplet chemotherapy, according to FOLFOXIRI plus BEV and FIr-B/FOx, consisting of 5-fluorouracil associated to alternating irinotecan/BEV or oxaliplatin, according to previously reported weekly schedule [5], increased activity and efficacy of MCRC patients: objective response rate (ORR) 77–82%, PFS 13.1 and 12 months, OS 30.9 and 28 months, not significantly different in *KRAS*_*2*_ wt and mut patients [5, 3]. Median OS of patients treated with FIr-B/FOx in *KRAS*_*2*_ wt and mut patients was 38 months and 21 months, respectively, not significantly different [9]; the prevalent *KRAS*_*2*_ c.35 G>A (G12D) mutant genotype may significantly affect worse OS [10, 11]. More recently, *KRAS*_*2*_ genotype was reported as affecting significantly different PFS and OS in patients treated with XelOx/BEV [21]. Retrospective analysis of clinical outcome according to *KRAS*_*2–4*_/*NRAS*_*2–4*_/*BRAF*_*15*_ genotype in PRIME, FIRE-3, PEAK, TRIBE randomized trials showed that EGFR- and VEGF-inhibitors are more and equivalently effective in *KRAS*_*2–4*_/*NRAS*_*2–4*_ wt patients [16–18, 3]. In the TRIBE study, FOLFOXIRI-Bev may predict a favourable effect in *BRAF*_*15*_ mut patients, compared to FOLFIRI-BEV [3].

The present study evaluated the prevalence, individual distribution and prognostic relevance of *KRAS*_*2–4*_/*NRAS*_*2–4*_/*BRAF*_*15*_ mutations, detected by next generation sequencing, in MCRC patients treated with FIr-B/FOx intensive first line treatment.

## RESULTS

*KRAS*_*2–4*_/*NRAS*_*2–4*_/*BRAF*_*15*_ genotype was evaluated in 67 tumoral samples of 87 MCRC patients treated with FIr-B/FOx first line treatment (77%) (Table 1): 58 (86.6%) primary tumours, 9 (13.4%) metastatic sites (3 liver, 2 peritoneal carcinomatosis, 2 ovary, 1 local recurrence, and 1 lymph node); 60 (89.6%) obtained before first line metastatic treatment, 7 (10.4%) after first line treatment. Demographic and baseline features of evaluated patients were representative of the overall treated population [9].

**Table 1:** Distribution of tumoral samples of MCRC patients according to site (primary or metastatic) and timing of sampling (pre- or post-treatment)

	Tumoral Samples					
Timing of sampling referred to FIr-B/FOx treatment	Total		Primary tumor		Metastatic site	
	No.	%	No.	%	No.	%
**Total No.**	67	100	58	86.6	9	13.4
**Pre-treatment**	60	89.6	53	79.1	7	10.4
**Post-treatment**	7	10.4	5	7.5	2	3.0

Abbreviations: No., number.

Table 2 shows the prevalence of *KRAS*_*2–4*_/*NRAS*_*2–4*_/*BRAF*_*15*_ wild-type (wt) and mutant (mut) samples: in 63 patients evaluable for *KRAS*_*2–4*_/*NRAS*_*2–4*_/*BRAF*_*15*_ (94.0%), triple wt were 14 (22.2%), and mut 49 (77.8%); *KRAS*_*2–4*_ wt patients were 21 (33.3%), mut 42 (66.7%), 4 samples not evaluable for *KRAS*_*2–4*_ genotype, because the coverage of specific amplicons was not sufficient for accurate mutation detection; *KRAS*_*2*_ wt patients were 23 (36.5%), mut 40 (63.5%); *NRAS*_*2–4*_ wt patients were 56 (83.6%), mut 11 (16.4%); *BRAF*_*15*_ wt patients were 62 (92.5%), mut 5 (7.5%). *KRAS* exon 2 mutational status was discordant between previous monogenic evaluation and NGS data in 10 patients (15.9%): 5 (7.9%) evaluated as mut by monogenic assay were wt by NGS; 4 (6.3%) wt by monogenic assay showed a *KRAS* exon 2 mutation by NGS; a different *KRAS* exon 2 mutation was reported in 1 patient (1.6%).

**Table 2:** Prevalence of *KRAS*/*NRAS*/*BRAF* wild-type and mutant samples

	Samples	Patients/Samples	
	All	wild-type	mutant
	No. (%)	No. (%)	No. (%)
***KRAS***_***2-4***_**/*NRAS***_***2-4***_**/*BRAF***_***15***_	63 (94)	14 (22.2)	49 (77.8)
***KRAS***_***2-4***_	63 (94)	21 (33.3)	42 (66.7)
***KRAS***_***2***_	63 (94)	23 (36.5)	40 (63.5)
***NRAS***_***2-4***_	67 (100)	56 (83.6)	11 (16.4)
***BRAF***_***15***_	67 (100)	62 (92.5)	5 (7.5)

Table 3 shows the prevalence of mutant samples, with single and multiple mutations, according to the mutation detection criteria. Overall, 60 mutations were detected in 49 mut samples, range 1–3 mut (median 1.22 mut/mut sample) at >500×cov and >1% AF detection criteria: 43 (71.7%) at >1000×cov/>5% AF; 9 (15%) at >500×cov/>5% AF; 8 (13.3%) at >1000×cov/<5% AF. The distribution of mut samples according to the mutation detection criteria, >1000×cov and >5% AF, >500×cov and >5%, and >1000×cov and <5% AF was, respectively: in 49 *KRAS*_*2–4*_/*NRAS*_*2–4*_/*BRAF*_*15*_ mut (77.8%), 40 (63.5%), 8 (12.7%), 1 (1.6%); in 42 (66.7%) *KRAS*_*2–4*_ mut, 32 (50.8%), 9 (14.3%), 1 (1.6%); in 40 (63.5%) *KRAS*_*2*_ mut, 30 (47.6%), 9 (14.3%), 1 (1.6%); in 11 (16.4%) *NRAS*_*2–4*_ mut, 7 (10.4%), 1 (1.5%), 3 (4.5%); in 5 (7.5%) *BRAF*_*15*_ mut, 1 (1.5%) at >500×cov and >5% AF, 4 (6%) at >1000×cov and <5% AF.

**Table 3:** Prevalence of single and multiple *KRAS*/*NRAS*/*BRAF* mutations according to mutation detection criteria

		Mutations		Mutation detection criteria		
		Single	≥2	Coverage >1000 Allelic fraction >5%	Coverage 500-1000 Allelic fraction >5%	Coverage >1000 Allelic fraction >1<5%
	No. (%)	No. (%)	No. (%)	No. (%)	No. (%)	No. (%)
**Mutations**	60			43 (71.7)	9 (15.0)	8 (13.3)
***KRAS***_***2-4***_**/*NRAS***_***2-4***_**/*BRAF***_***15***_	49 (77.8)	41 (65.1)	8 (12.7)	40 (63.5)	8 (12.7)	1 (1.6)
***KRAS***_**2**_	40 (63.5)	33 (52.4)	7 (11.1)	30 (47.6)	9 (14.3)	1 (1.6)
***KRAS***_**2-4**_	42 (66.7)	35 (55.6)	7 (11.1)	32 (50.8)	9 (14.3)	1 (1.6)
***NRAS***_**2-4**_	11 (16.4)	6 (8.9)	5 (7.5)	7 (10.4)	1 (1.5)	3 (4.5)
***BRAF***_**15**_	5 (7.5)	0 (-)	5 (7.5)	0 (-)	1 (1.5)	4 (6.0)

The distribution of *KRAS*_*2–4*_/*NRAS*_*2–4*_/*BRAF*_*15*_ mutations, single and multiple, in individual mutant sample was, respectively: in 49 *KRAS*_*2–4*_/*NRAS*_*2–4*_/*BRAF*_*15*_ mut samples, 41 single (65.1%) and 8 (12.7%) ≥2 mut; in 42 *KRAS*_*2–4*_ mut, 35 (55.6%) single and 7 (11.1%) ≥2 mut; in 40 *KRAS*_*2*_ mut, 33 (52.4%) single and 7 (11.1%) ≥2 mut; in 11 *NRAS*_*2–4*_ mut, 6 (8.9%) single and 5 (7.5%) ≥2 mut; in 5 *BRAF*_*15*_ mut, all ≥2 mut (7.5%).

Table 4 shows the panel of detected mutations, and of the >2 mut samples according to mutation detection criteria. Distribution of *KRAS*_*2*_ mut was: codon 12, 35 (55.6%); codon 13, 5 (7.9%). *KRAS*_*2*_ c.35 G>A (G12D) was detected in 16 (25.4%) samples, 13 (20.6%) single and 3 (4.8%) ≥2 mut: 1 associated with *NRAS*_*2*_ c.34 G>A (G12S) and *BRAF*_*15*_ c.1756 G>A (E586K) mutations; 1 associated with *NRAS*_*2*_ c.38 G>T (G13V) and *BRAF*_*15*_ c.1405 G>A (G469R) mutations; 1 associated with *NRAS*_*2*_ c.182 A>G (Q61R) mutation. *KRAS*_*2*_ c.35 G>T (G12V) was detected in 15 (23.8%) samples, 13 (20.6%) single and 2 (3.2%) ≥2 mut: 1 associated with a *KRAS*_*4*_ c.436 G>A (A146T) mutation; 1 associated with a *BRAF*_*15*_ c.1756 G>A (E586K) mutation. Other *KRAS*_*2*_ mutations were: c.35 G>C (G12A) 1 (1.6%); c.34 G>C (G12R) 1 (1.6%); c.34 G>A (G12S) 2 (3.2%), including 1 associated with *KRAS* c.37 G>A (G13S) and *NRAS* c.35 G>A (G12D); c.38 G>A (G13D), 5 (7.9%), 4 (6.4%) single and 1 (1.6%) ≥2 mut, associated with a *BRAF*_*15*_ c.1406 G>C (G469A) mutation. *KRAS*_*3*_ codon 61 mut were detected in 2 patients (3.2%), specifically c.182 A>T (Q61L) and c.182 A>G (Q61R), 1 patient each (1.6%). Distribution of *NRAS* mutations was: *NRAS* codon 12, 2 (3.0%), specifically c.34 G>A (G12S) associated to *KRAS* c.35 G>A and *BRAF* c.1756 G>A mutations, and c.35 G>A (G12D) associated to double *KRAS* mutations; *NRAS* codon 13, 4 (6%), c.37 G>A (G13S) 1 patient (1.5%), c.38 G>A (G13D) 2 patients (3%), and c.38 G>T (G13V) associated to *KRAS* c.35 G>A and *BRAF* c.1405 G>A mutations, 1 patient (1.5%); *NRAS* codon 61, 5 patients (7.5%), specifically c.182 A>T (Q61L) 1 (1.5%), and c.182 A>G (Q61R) 4 (6%) patients, 2 single and 2 associated to *KRAS* c.35 G>A, and to *BRAF* c.1796 C>T (T599I) mutations, respectively. All 5 (7.5%) *BRAF*_*15*_ mut were atypical and associated to *KRAS* and/or *NRAS* mutations: c.1405 G>A (G469R) associated to *KRAS* c.35 G>A and *NRAS* c.38 G>T mutations; c.1406 G>C (G469A) associated to *KRAS* c.38 G>A mutation; c.1756 G>A (E586K) 2 samples, associated to *KRAS* c.35 G>A/*NRAS* c.34 G>A, and to *KRAS* c.35 G>T mutations, respectively; c.1796 C>T (T599I) associated to *NRAS* c.182 A>G mutation.

**Table 4:** Panel of single and multiple *KRAS*/*NRAS*/*BRAF* mutations in individual mutant samples, according to mutation detection criteria

		Mutations		≥ 2 mutant samples			Mutation detection criteria		
		1	≥2	*KRAS*	*NRAS*	*BRAF*	Cov >1000 AF >5%	Cov 500-1000 AF >5%	Cov >1000 AF >1<5%
	No. (%)	No. (%)	No. (%)	No.	No.	No.	No. (%)	No. (%)	No. (%)
***KRAS***									
35 G>A	16 (25.4)	13 (20.6)	3 (4.8)	35 G>A 35 G>A 35 G>A	34 G>A 38 G>T 182 A>G	1756 G>A 1405 G>A	2 1 1	0 0 0	1 (*BRAF*) 2 (*NRAS*,*BRAF*) 1 (*NRAS*)
35 G>T	15 (23.8)	13 (20.6)	2 (3.2)	35 G>T + 436 G>A 35 G>T		1756 G>A	1 1	0 0	1 (*KRAS*) 1 (*BRAF*)
35 G>C	1 (1.6)	1 (1.6)	0 (-)						
34 G>C	1 (1.6)	1 (1.6)	0 (-)						
34 G>A	2 (3.2)	1 (1.6)	1 (1.6)	34 G>A + 37 G>A	35 G>A		3	0	0
38 G>A	5 (7.9)	4 (6.4)	1 (1.6)	38 G>A		1406 G>C	1	1	0
182 A>T	1 (1.6)	1 (1.6)	0 (-)						
182 A>G	1 (1.6)	1 (1.6)	0 (-)						
***NRAS***									
37 G>A	1 (1.5)	1 (1.5)	0 (-)						
38 G>A	2 (3.0)	2 (3.0)	0 (-)						
182 A>T	1 (1.5)	1 (1.5)	0 (-)						
182 A>G	4 (6.0)	2 (3.0)	2 (3.0)		182 A>G	1796 C>T	0	0	2 (*NRAS*,*BRAF*)

Abbreviations: Cov, coverage; AF, allelic fraction.

All the 8 mutations detected at >1000×cov and <5% AF (13.3%) and consisting of 1 *KRAS,* 3 *NRAS,* 4 *BRAF* mutations, were detected in 6 mutant samples harboring ≥2 mutations: 5 associated to *KRAS* mutations detected at >1000×cov and >5% AF; 1 (1.6%) mut sample with a *NRAS* c.182 A>G (Q61R) associated to *BRAF* c.1796 C>T (T599I) mutation represented the only sample with mutations detected at >1000×cov and <5% AF.

### Efficacy according to *KRAS*/*NRAS*/*BRAF* genotype and specific mutations

**Figure 1:**
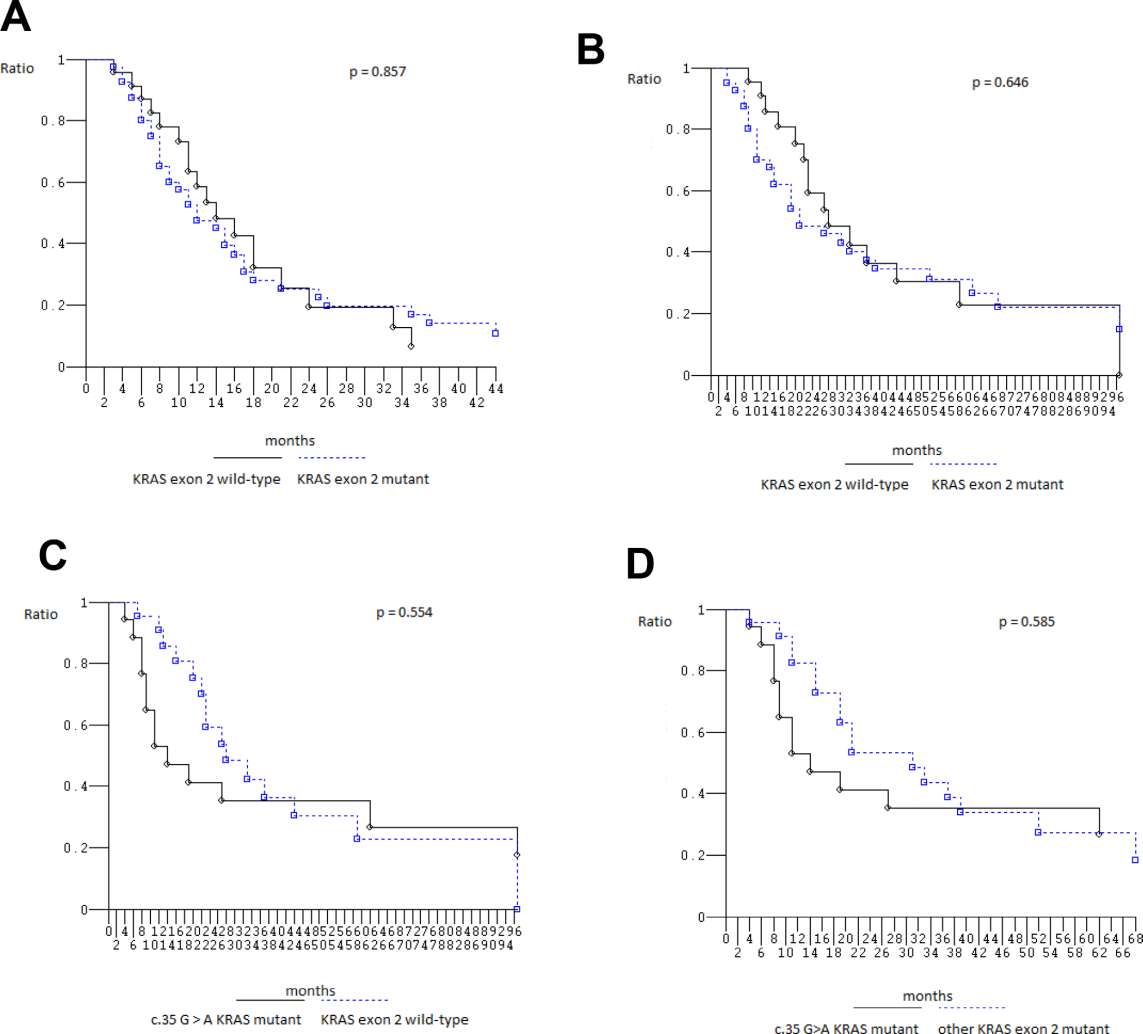
Kaplan–Meier survival estimate (**A**) Progression-free Survival *KRAS* exon 2 wild-type versus mutant; (**B**) Overall Survival *KRAS* exon 2 wild-type versus mutant; (**C**) Overall Survival c.35 G > A *KRAS* mutant patients versus *KRAS* exon 2 wild-type patients; (**D**) Overall Survival c.35 G > A *KRAS* mutant patients versus other *KRAS* exon 2 mutant patients.

**Figure 2:**
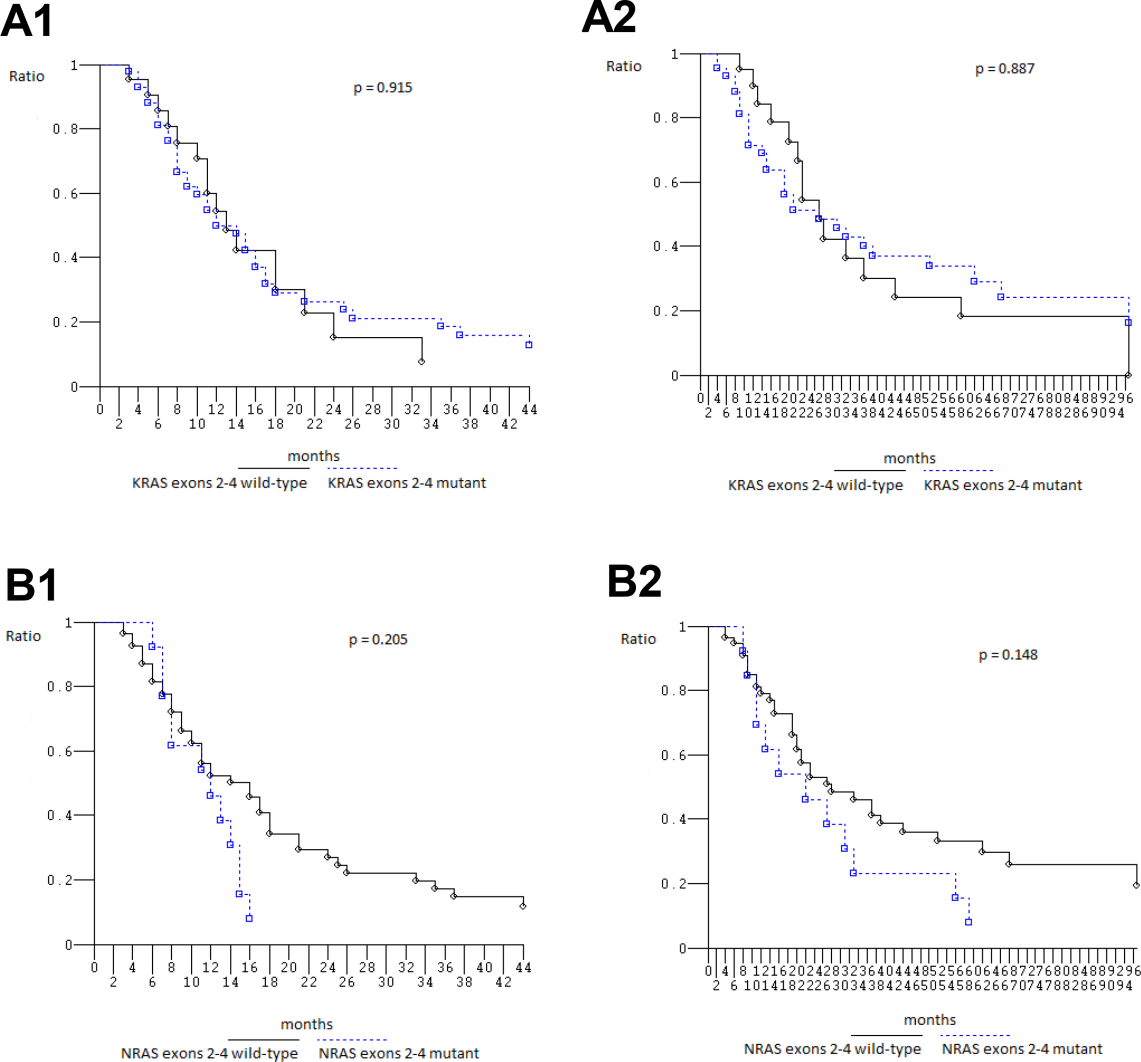
Overall Survival, Kaplan–Meier survival estimate (**A**) *KRAS*_2-4_ wild type versus mutant; (**B**) *NRAS*_2-4_ wild-type versus mutant; 1, Progression-free survival; 2, Overall survival.

**Figure 3:**
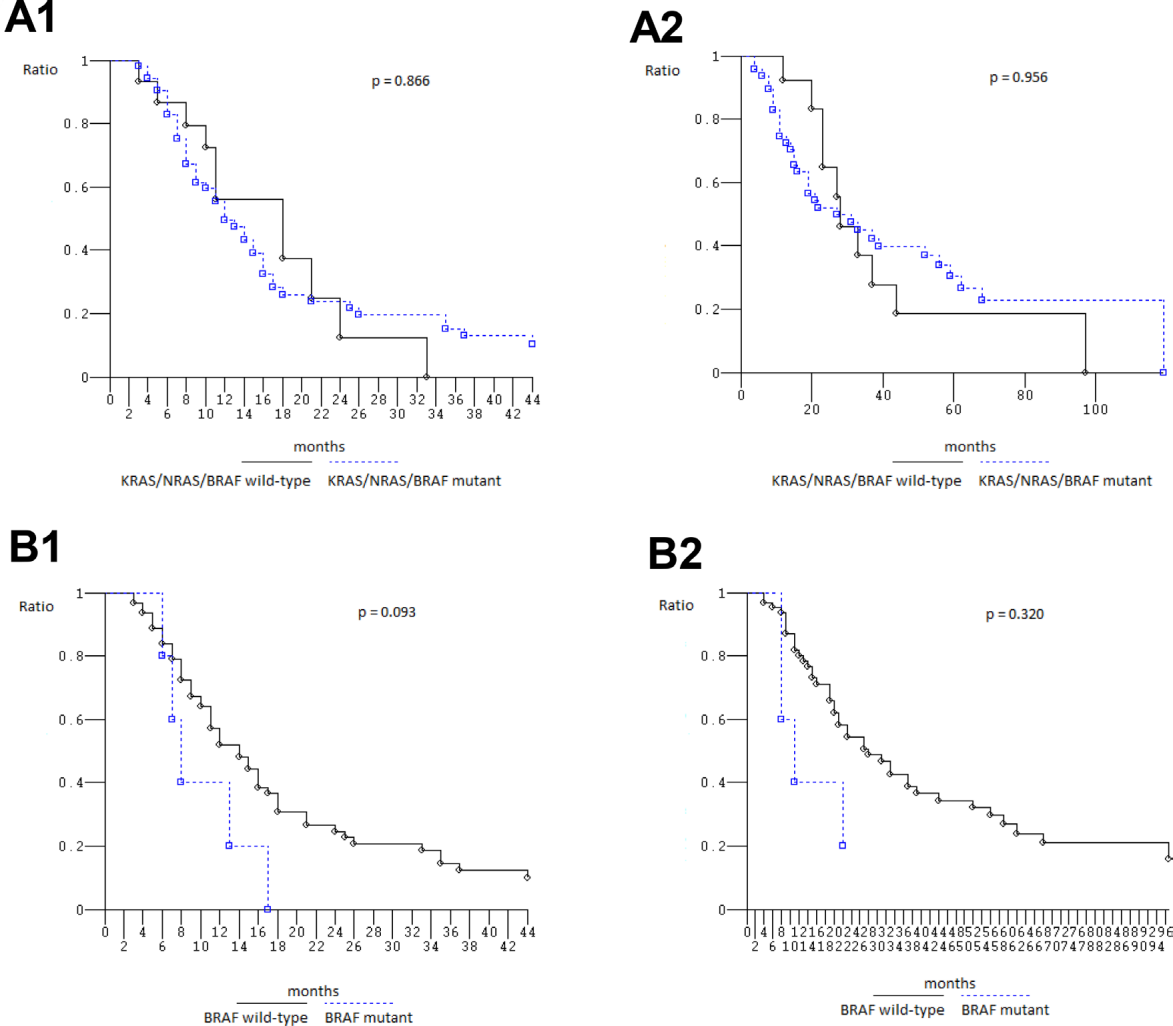
Overall Survival, Kaplan–Meier survival estimate (**A**) *KRAS*/*NRAS*/*BRAF* wild-type versus mutant patients; (**B**) *BRAF* wild-type versus *BRAF* mutant patients; 1, Progression-free survival; 2, Overall survival.

At median follow-up 21 months, clinical outcome of 67 patients evaluated for *KRAS*_*2–4*_/*NRAS*_*2–4*_/*BRAF*_*15*_ genotype was consistent with previously reported in overall population treated with FIr-B/FOx intensive first line (Table 5) [9, 10, 22]: PFS 13 months (3–113+ months), OS 27 months (4–119+ months); in *KRAS*_*2*_ wt and mut, median PFS was 14 (3–93+ months) and 12 months (3–113+ months), median OS 28 (6+−97 months) and 21 months (4–119+ months), respectively, not significantly different [23] (Figure 1A, 1B); in c.35 G>A *KRAS* mut, median PFS was 8 months (3–113+ months), median OS 14 months (4–119+ months), not significantly different compared with wt (Figure 1C, 1D). Among *KRAS*_*2–4*_ wt and mut, median PFS was 13 (3–93+ months) and 12 months (3–113+ months), median OS 27 months equivalently (6+−97 and 4–119+ months), respectively, not significantly different (Figure 2A). Among *NRAS*_*2–4*_ wt and mut, median PFS was 16 (3–113+ months) and 12 months (6–93+ months), median OS 28 (4–119+ months) and 22 months (8–93+ months), respectively, not significantly different (Figure 2B). Among *BRAF*_*15*_ wt and mut, median PFS was 14 (3–113+ months) and 8 months (6–17 months), median OS was 28 (4–119+ months) and 11 months (8–94+ months), respectively (Figure 3A), not significantly different. Among *KRAS*_*2–4*_/*NRAS*_*2–4*_/*BRAF*_*15*_ wt and mut, median PFS was 18 (3–33 months) and 12 months (3–113+ months), median OS was 28 (6+−97 months) and 22 months (4–119+ months), respectively (Figure 3B). PFS and OS of wt compared to mut patients was not significantly different (*P* = 0.866 and 0.956, respectively). Among mutant patients with ≥2 compared to 1 mut gene, PFS was 11 and 14 months, OS was 22 and 37 months, not significantly different. PFS was trendy worse in patients harboring ≥2 mut *KRAS*_*2–4*_/*NRAS*_*2–4*_/*BRAF*_*15*_ genes compared with triple wt (*P* 0.059).

**Table 5:** Efficacy according to *KRAS*/*NRAS*/*BRAF* genotype

	Patients		Progression-free survival (months) Range	Overall survival (months) Range
	No.	%		
**Overall evaluated population**	67	100	13 3–113+	27 4–119+
***KRAS* exon 2 wild-type**	23	37.5	14 3–93+	28 6+−97
***KRAS* exon 2 mutan**t	40	63.5	12 3–113+	21 4–119+
**c.35 G > A *KRAS* mutant**	16	25.3	8 3–113+	14 4–119+
***KRAS* exon 2-4 wild-type**	21	33.3	13 3–93+	27 6+−97
***KRAS* exon 2-4 mutant**	42	66.7	12 3–113+	27 4–119+
***NRAS* exon 2-4 wild-type**	56	83.6	16 3–113+	28 4–119+
***NRAS* exon 2-4 mutant**	11	16.4	12 6–93+	22 8–93+
***BRAF* wild-type**	62	92.5	14 3–113+	28 4–119+
***BRAF* mutant**	5	7.5	8 6–17	11 8–94+
***KRAS*/*NRAS*/*BRAF* wild-type**	14	22.2	18 3–33	28 6+−97
***KRAS*/*NRAS*/*BRAF* mutant**	49	77.8	12 3–113+	22 4–119+
**Single mutant gene**	40	63.5	14 3–113+	37 4–19+
**≥2 mutant genes**	9	14.3	11 6–17	22 8–94+

Abbreviations: No, number.

Among 22 L-L (32.8%) patients, median PFS was 16 months (3–113+), median OS 33 months (6+−113+): in *KRAS*_*2–4*_/*NRAS*_*2–4*_/*BRAF*_*15*_ wt and mut, median PFS was 18 (3–33 months) and 16 months (5–113+ months), median OS was 33 (6+-97 months) and 39 months (8–113+ months), respectively, not significantly different (*P* 0.685 and 0.480 respectively). Among 45 O/MM patients (67.2%), median PFS was 12 months (3–59+), median OS 23 months (4–119+): in *KRAS*_*2–4*_/*NRAS*_*2–4*_/*BRAF*_*15*_ wt and mut, median PFS was 18 (5–24 months) and 12 months (3–59+ months), median OS was 28 (10+-70+ months) and 21 months (4–119+ months), respectively, not significantly different (*P* 0.624 and 0.538 respectively). Among 13 right side patients (19.4%), median PFS was 14 months (5–113+), median OS 20 months (6+−113+): only 1 patient was *KRAS*_*2–4*_/*NRAS*_*2–4*_/*BRAF*_*15*_ wt with PFS 11 months and OS 44 months; among *KRAS*_*2–4*_/*NRAS*_*2–4*_/*BRAF*_*15*_ mut median PFS 16 months (5–113+) and median OS 19 months (6–113+). Among 54 left side patients (81.6%), median PFS was 13 months (3–88+), median OS 27 months (4–119+); among *KRAS*_*2–4*_/*NRAS*_*2–4*_/*BRAF*_*15*_ wt median PFS 18 months (3–33) and OS 28 months (6+−97); among *KRAS*_*2–4*_/*NRAS*_*2–4*_/*BRAF*_*15*_ mut median PFS 12 months (3–88+) and median OS 27 months (4–119+). In the overall analysed population, PFS and OS of right compared with left tumors were not significantly different (*P* 0.675 and 0.751, respectively).

## DISCUSSION

The present study evaluated the prevalence and individual distribution of *KRAS*_*2–4*_/*NRAS*_*2–4*_/*BRAF*_*15*_ mutations by NGS in MCRC patients treated with intensive first line treatment adding BEV to triplet chemotherapy according to previously reported FIr-B/FOx schedule [5]. Next generation sequencing by Ion Torrent platform, mostly performed before first line metastatic treatment (89.6% of samples) and in primary tumor samples (79.1%), detected overall 77.8% *KRAS*_*2–4*_/*NRAS*_*2–4*_/*BRAF*_*15*_ mut MCRC patients at molecular diagnostic criteria of target sequence coverage >500×cov and >1% mutant AF; specifically, *KRAS*_*2–4*_ 66.7%, *NRAS*_*2–4*_ 16.4%, *BRAF*_*15*_ 7.5% mut MCRC patients. *KRAS*_*2*_ and *KRAS*_*2–4*_/*NRAS*_*2–4*_/*BRAF*_*15*_ mut at >1000×cov and >5% AF were 47.6% and 63.5%, respectively, consistent with that previously reported in the same MCRC cohort by our group using direct sequencing [9, 10], and in the range of reported 45–55% *KRAS*_*2*_ and 65.6% *KRAS*_*2–4*_/*NRAS*_*2–4*_/*BRAF*_*15*_ mut [3]. In the retrospective evaluation of TRIBE trial [3], *KRAS*_*2–4*_, *NRAS*_*2–4*_*, BRAF*_*15*_ mutations were detected in 52.8%, 5.3% and 7.5% of patients, respectively; all wild-type were 34.4%. The application of >500×cov and >1% AF mutation criteria may increase detectable mutations due to the increased NGS diagnostic accuracy, that may detect tumoral clonal heterogeneity: *KRAS*_*2–4*_ mut were prevalently detected at >1000×cov/>5% AF (50.8%), while *NRAS*_*2–4*_ mut (16.4%) were frequently detected at 500–1000xcov and <5%AF (6%), and *BRAF*_*15*_ mut (7.5%) were all detected at 500–1000×cov and <5%AF. Thus*, KRAS*_*2–4*_/*NRAS*_*2–4*_/*BRAF*_*15*_ mutations were frequently detected (28.3%) at 500–1000×cov and <5%AF.

Present findings show that *RAS* mutations were enriched in MCRC patients, compared to early CRC where *KRAS*_*2–4*_ 37.8%, and *NRAS*_*2–4*_ 4.6% mut were reported by the same NGS platform [13, 14].

*KRAS*_*2–4*_/*NRAS*_*2–4*_/*BRAF*_*15*_ mut were often detected at <5% AF (13.3%), all involving MCRC patients harbouring >2 *KRAS* and/or *NRAS* mut and prevalently involving atypical *BRAF* mutations, different from that typically reported (codon 600) in CRC [14]. All *BRAF*_*15*_ mut harboured >2 mut, associated with other *KRAS*_*2–4*_/*NRAS*_*2–4*_ mut, all atypical. More, *KRAS*, *NRAS, BRAF* mutations were not mutually exclusive: tumoral samples prevalently harboured single gene (*KRAS*_*2–4*_, *NRAS*_*2–4*_) mutations (65.1%); individual MCRC patients often harboured double or triple *KRAS*_*2–4*_/*NRAS*_*2–4*_/*BRAF*_*15*_ mutations (12.7%), frequently involving *NRAS*_*2–4*_ and all *BRAF*_*15*_ mut, as recently reported [13, 14]. One MCRC patient was only detected at 500–1000×cov and <5%AF (1.6%), and harbored *NRAS* c.182 A>G (Q61R) associated to *BRAF* c.1796 C>T (T599I) mutations. Thus, massive parallel sequencing by Ion torrent platform can increase mutation detection by increasing diagnostic accuracy, if >1% mutant AF with >1000×cov is included to specifically detect clonal heterogeneity involving *KRAS*, *NRAS* and atypical *BRAF* mutations, thus increasing the detection of multiple genes mutations in individual MCRC patients. To this aim, NGS is able to detect mutant alleles at the 5% level [13, 14].

Specific mutations of different genes involved in the same signalling pathway (*BRAF* and *RAS* mutations) can confer different biological aggressiveness and effectiveness of treatment strategies. Preliminary analysis of differential clinical outcome in overall MCRC patients treated with FIr-B/FOx intensive first line treatment according to *KRAS*_*2*_ genotype confirmed previously reported median PFS 13 months and OS 27 months, a trendy worse OS 21 months in *KRAS*_*2*_ mut, and PFS 8 months and OS 14 months in the prevalent *KRAS*_*2*_ c.35G > A mut MCRC patients [11]. Clinical outcome was not significantly different in *KRAS*_*2–4*_, *NRAS*_*2–4*_*, BRAF*_*15*_ mut and wt MCRC patients. The 5 *BRAF*_*15*_ mut, all atypical and associated to other *KRAS*_*2–4*_ and/or *NRAS*_*2–4*_ mut, compared to wt MCRC patients showed trendy worse, not significantly different, PFS 8 months and OS 11 months, even if treated with BEV added to triplet chemotherapy. Worse prognosis was previously shown by the prevalent *BRAF*_*15*_ c.1799 T > A (V600E) mutation, characterizing 4.7–8.7% CRC, in MCRC patients treated with doublet chemotherapy alone or added to cetuximab, BEV, and cetuximab plus BEV, with median PFS 5.6–8 months and median OS 10.3–15.9 months [24–26]. The favourable predictive effect of cetuximab or BEV addiction to chemotherapy in *KRAS* exon 2 wild-type patients was not significantly confirmed in *BRAF* mutant MCRC patients [20, 24, 25]. Mutations in *BRAF* gene occur in two regions of the BRAF kinase domain, exon 15, the activation segment (which protects the substrate binding site), and, less commonly, exon 11, the G loop (which mediates ATP-binding). Less common *BRAF* mutations at codons 594 and 596 correlated with longer OS when compared with *BRAF* V600E mutations (62 vs 12.6 months, *P* = 0.002) [27], and trendy longer compared to *BRAF* wild-type (35.9 months) in MCRC patients treated with FOLFOXIRI/BEV [3].

Differential clinical outcome according to *KRAS*_*2–4*_/*NRAS*_*2–4*_/*BRAF*_*15*_ genotype mut and wt was not significantly different (PFS 12 vs 18 months; OS 22 vs 28 months). The retrospective evaluation of TRIBE trial also reported no significant interaction between *RAS* or *BRAF* status and treatment effect in PFS or OS [3]. *RAS* mutant patients treated with first line FOLFOXIRI plus BEV achieved median OS 25.6 months, *BRAF* mutant 13.4 months, and triple wild-type 37.1 months.

In the 22.2% *KRAS*_*2–4*_/*NRAS*_*2–4*_/*BRAF*_*15*_ triple wt MCRC patients, the efficacy was trendy higher with a median PFS 18 months and median OS 28 months; furthermore, the efficacy of first line FIr-B/FOx, adding BEV to triplet chemotherapy is close to be significantly different in *KRAS*_*2–4*_/*NRAS*_*2–4*_/*BRAF*_*15*_ MCRC triple wt compared with MCRC patients harbouring ≥2 mutant genes (PFS 11 months) and it requires further prospective validation.

Prospective studies should be developed to better evaluate differential clinical outcome in MCRC patients harbouring *KRAS* c.35 G > A (G12D), *BRAF* c.1799 T > A (V600E) and atypical, less common mutations, as well as in *KRAS*_*2–4*_/*NRAS*_*2–4*_/*BRAF*_*15*_ wt and in patients harbouring ≥2 mutant genes.

More, high sensitive *KRAS*_*2–4*_/*NRAS*_*2–4*_/*BRAF*_*15*_ multigenic analysis performed in metastatic tissues and/or liquid biopsies could dynamically be helpful to monitor the evolution of mutant genes spectrum, more closely evaluate prognostic implications and individually predict targeted treatment.

## MATERIALS AND METHODS

### Patients and samples

Eighty-seven consecutive, unselected, MCRC patients were enrolled in previously reported phase II study and the expanded clinical program proposing FIr-B/FOx as first line treatment [5, 9]. *KRAS*_*2–4*_/*NRAS*_*2–4*_/*BRAF*_*15*_ genotype was evaluated in tumoral samples of 67 (77%) patients (Table 1), specifically primary tumours or metastatic sites, pre- or post-treatment.

Study was approved by Local Ethical Committee (Comitato Etico, Azienda Sanitaria Locale n.4 L’Aquila, Regione Abruzzo, Italia) and conducted in accordance with Declaration of Helsinki. All patients provided written, informed consent.

FIr-B/FOx association consisted of 5-fluorouracil associated to alternating irinotecan/BEV or oxaliplatin, according to previously reported weekly schedule [5].

### Mutational analysis

*KRAS*_*2–4*_/*NRAS*_*2–4*_/*BRAF*_*15*_ analyses were performed on paraffin-embedded tissue blocks from primary tumor and/or metastatic site. Genotype status was previously analyzed for *KRAS*_*2*_ (codon 12–13) mutations and *BRAF* c.1799 T>A (V600E) mutations, according to genotype analyses recommendations for clinical implications [28], by SNaPshot^®^ multiplex, and/or direct sequencing, as previously reported [9].

### Mutation detection by massive parallel sequencing

Neoplastic cells were selected from highest density H&E slide; DNA was extracted by QIAamp Mini Kit from 2–5 10 μm serial sections, assessed by Qubit photometer, dsDNA High Sensitivity Assay Kit. Workflow consisted of generation of library of DNA amplified fragments flanked by Ion Torrent adapters, clonally amplified into Ion Sphere particles (ISP) by emulsion PCR, applied to Ion chip on Ion PGM sequencer and analyzed (Life Technologies): 10 ng DNA for library preparation with Ion AmpliSeq Library 96LV Kit 2.0, Colon/Lung Cancer Panel, including 207 amplicons covering 2800 hotspot regions in 50 genes with >500×sequence coverage; library barcoded with Ion Xpress Barcode Adapters 1–16 Kit; template prepared by emulsion PCR on Ion OneTouch 2; library quality control performed by Ion Sphere Quality Control Kit; sequencing primer, and polymerase added and loaded onto 316 chips; sequencing performed on PGM, data analysis with Torrent Suite Software V.3.2. Variant Caller plug-in applied by Colon/Lung hotspot file (http://www.thermofisher.com); Ion Reporter suite used to filter polymorphic variants, reviewed with Integrative Genomics Viewer (IGV V.2.1 Broad Institute Cambridge Massachusetts USA) [13, 14]. Molecular diagnostic criteria for mutation detection were: >500×sequence coverage; >1% mutant allelic fraction.

### Study design

A retrospective analysis has been planned to evaluate prognostic relevance of *KRAS*_*2–4*_**/***NRAS*_*2–4*_**/***BRAF*_*15*_ genotype on clinical outcome of MCRC patients treated with first line FIr-B/FOx regimen. Clinical criteria of efficacy were PFS, OS, evaluated using Kaplan and Meier method [22]. Patients were evaluated according to involved metastatic sites, classified as L-L and O/MM. Log-rank test was used to compare PFS and OS in different subgroups [23]. PFS was defined as length of time between beginning of treatment and disease progression or death (resulting from any cause) or to last contact; OS as length of time between beginning of treatment and death or to last contact.

## CONCLUSIONS

Next generation sequencing of multiple genes shows that most MCRC harbour *KRAS*_*2–4*_/*NRAS*_*2–4*_/*BRAF*_*15*_ mutations, prevalently as single gene mut, and frequently multiple gene mutations, at increased sensitivity due to clonal heterogeneity.

Clinical outcome of MCRC patients treated with intensive first line FIr-B/FOx regimen was not significantly affected by *KRAS*_*2–4*_/*NRAS*_*2–4*_/*BRAF*_*15*_ genotype status.

Differential prognosis and predictive effect of VEGF-inhibitors added to chemotherapy in MCRC patients harbouring *KRAS*_*2*_ c.35 G > A and *BRAF*_*15*_ mut, or *KRAS*_*2–4*_/*NRAS*_*2–4*_/*BRAF*_*15*_ wt should be prospectively evaluated by massive parallel sequencing, also according to other mutations differentially activating the downstream RAS-MAPK or PI3K pathways.

## Abbreviations

AF: allelic fraction
anti-EGFR: anti-epidermal growth factor receptor
anti-VEGF: anti-vascular endothelial growth factor
BEV: bevacizumab
*BRAF*_15_: *BRAF* exon 15
cov: coverage
CRC: colorectal cancer
HR: hazard ratio
*KRAS*_2–4_: *KRAS* exons 2–4
IFL: irinotecan, 5-fluorouracil, and leucovorin
ISP: Ion Sphere particles
L-L: liver-limited
m: months
(MAPK): mitogen-activated protein kinase
MCRC: metastatic colorectal cancer
mut: mutant
NGS: Next Generation Sequencing
*NRAS*_2–4_: *NRAS* exons 2–4
O/MM: other multiple metastatic sites
ORR: objective response rate
OS: overall survival
PFS: progression-free survival
(PI3K): phosphoinositide 3-kinase
wt: wild-type
